# Biological knowledge-slanted random forest approach for the classification of calcified aortic valve stenosis

**DOI:** 10.1186/s13040-021-00269-4

**Published:** 2021-07-23

**Authors:** Erika Cantor, Rodrigo Salas, Harvey Rosas, Sandra Guauque-Olarte

**Affiliations:** 1 Institute of Statistics, Universidad de Valparaíso, Valparaíso, Chile; 2 School of Biomedical Engineering, Universidad de Valparaíso, Valparaíso, Chile; 3 Centro de Investigación y Desarrollo en Ingeniería en Salud, CINGS-UV, Universidad de Valparaíso, Valparaíso, Chile; 4 Faculty of Dentistry, Universidad Cooperativa de Colombia, Envigado, Colombia

**Keywords:** Machine learning, Calcific aortic valve disease, Random Forest, Prior-knowledge, Gene-selection

## Abstract

**Background:**

Calcific aortic valve stenosis (CAVS) is a fatal disease and there is no pharmacological treatment to prevent the progression of CAVS. This study aims to identify genes potentially implicated with CAVS in patients with congenital bicuspid aortic valve (BAV) and tricuspid aortic valve (TAV) in comparison with patients having normal valves, using a knowledge-slanted random forest (RF).

**Results:**

This study implemented a knowledge-slanted random forest (RF) using information extracted from a protein-protein interactions network to rank genes in order to modify their selection probability to draw the candidate split-variables. A total of 15,191 genes were assessed in 19 valves with CAVS (BAV, *n* = 10; TAV, *n* = 9) and 8 normal valves. The performance of the model was evaluated using accuracy, sensitivity, and specificity to discriminate cases with CAVS. A comparison with conventional RF was also performed. The performance of this proposed approach reported improved accuracy in comparison with conventional RF to classify cases separately with BAV and TAV (Slanted RF: 59.3% versus 40.7%). When patients with BAV and TAV were grouped against patients with normal valves, the addition of prior biological information was not relevant with an accuracy of 92.6%.

**Conclusion:**

The knowledge-slanted RF approach reflected prior biological knowledge, leading to better precision in distinguishing between cases with BAV, TAV, and normal valves. The results of this study suggest that the integration of biological knowledge can be useful during difficult classification tasks.

**Supplementary Information:**

The online version contains supplementary material available at 10.1186/s13040-021-00269-4.

## Introduction

Calcific aortic valve stenosis (CAVS) is one of the main causes of morbidity and mortality in the elderly. In cases with CAVS, a restriction of blood flow occurs attributed to the narrowing of the aortic valve between the left ventricle and the aorta. The incidence of CAVS is strongly related to age, ranging from 0.2 to 9.8% between the fifth and eighth decade of life [1]. Although a normal aortic valve has three leaflets, a congenital bicuspid aortic valve (BAV) composed of two leaflets is found in approximately 1–2% of the population [2]. Patients with BAV and tricuspid aortic valve (TAV) are susceptible to develop CAVS and its etiology can be classified as congenital or degenerative associated with a chronic process by progressive mineralization. Currently, no conservative treatment is available to prevent the progression of CAVS and valve replacement is still the only treatment option to treat severe cases [3]. Consequently, the identification of candidate genes that are relevant in the CAVS process is imperative to improve the understanding of the mechanisms behind calcified BAV and TAV and discover potential medical treatments.

On the other hand, differential gene expression analysis from RNA-sequencing (RNA-Seq) experiments is the most common statistical analysis to reveal differences in gene expression levels between samples. Generally, the identification and selection of differentially expressed genes have been carried out using hypothesis testing through statistical models based on a Poisson distribution or a Negative Binomial distribution. This conventional approach performs a univariate statistical test for each gene, which can lead to the identification of thousands of genes with small effects, and thus, this approach could become increasingly difficult [4, 5].

In the last few years, machine learning (ML) techniques have been applied to several genetic problems to analyze the large amount of data allowing the simultaneous manipulation of hundreds to thousands of genes, although their results can be difficult to interpret [6]. Random forest (RF) algorithm is one of the commonly used tree ensemble methods in genomic high-dimensional datasets due to its ability to capture non-linear relationships, handle categorical and continuous variables and allow integration of information from multiple data sources [7]. RF has been successfully implemented in prediction applications using omic variables (e.g, gene and protein expression, single nucleotide polymorphisms), pathway analysis [8], or reconstruction of protein-protein interactions [9].

The potential of ML and RF to select important genes related to particular conditions has been recognized in life science [7, 10], but they are considered as black-box models, hindering decisions making based on their results. For this reason, the involvement of prior knowledge encapsulated through networks that describe gene-gene interaction has been explored, improving the performance of the models [11–14].

Accordingly, the objective of this study was to identify genes potentially implicated with CAVS in patients with congenital bicuspid aortic valve (BAV) and tricuspid aortic valve (TAV) in comparison with patients having normal valves. In this article, we implemented a knowledge-slanted random forest (RF) using information extracted from a protein-protein interactions (PPI) network to rank genes. For this, a random walk with restart (RWR) algorithm was used to determine the relevance of each gene based on its connection and localization with respect to other genes. We explored how the use of biological knowledge can improve RF performance in classification tasks. Furthermore, not many studies have compared the gene expression profile of BAV and TAV patients in order to identify gene targets differentiating the development or progression of CAVS with respect to aortic valve configuration.

## Methods

### Medical dataset used

We analyzed 27 men with a mean age of 62.6 ± 4.7 years. Cases with BAV (*n* = 10), TAV (*n* = 9), and controls without evidence of CAVS with a normal aortic valve function (*n* = 8) were included. Selection criteria consisted of patients with BAV/TAV who underwent aortic valve replacement with a valve fibro-calcific remodeling score of 3 and without type 2 diabetes, renal insufficiency, or ascending aorta replacement. Patients with CAVS were matched by age ± 10 years with respect to the controls who were selected because they underwent orthotopic heart transplantation without CAVS. All procedures were performed between 2005 and 2011 at the Institut universitaire de cardiologie et de pneumelogie de Québec. RNA extraction was performed from one leaflet of normal and CAVS valves. Specified details about clinical and echocardiographic characteristics of patients, tissue description, RNA extraction, and RNA sequencing can be consulted in Guauque-Olarte et al. [15]. Finally, this dataset consisted of expression levels of 15,191 genes from RNA-seq in 27 samples. Expression counts were normalized using the trimmed mean of M values “TMM”.

### PPI network and gene prioritization

Prior knowledge represented through a PPI network is relevant because the genes associated with a specific disease share similar functions and tend to be located in neighboring regions on the PPI network, which helps to identify new disease-related genes and perform the candidate-gene prioritization [16]. In this study, a PPI network was downloaded from the STRING website (https://string-db.org/), which reports for each gene-gene interaction a score from 0 to 1 as a measure of confidence that the reported interaction is true given the available evidence [17]. An undirected weighted graph *G* = (*V*, *E*) is retrieved, where nodes *i*, *j* ∈ *V* correspond to each gene, and edges or connections (*i*, *j*) ∈ *E* are weighted with a weight matrix *W* created using the scores from STRING. Finally, the resulting PPI network contained the information of 15,191 nodes (genes).

For gene prioritization, an RWR algorithm was applied to rank the genes on the PPI network [18]. RWR simulates a random walker that explores the PPI network from node *i* to node *j* using a transition probability matrix *A* = *D*^−1^*W*, where *D* is a diagonal matrix with elements *d*_*ij*_ = ∑_*j*_*w*_*ij*_. In addition, the random walker can move from *i* node to a randomly neighbor node or goes back to the initial node with a back-probability *θ* ∈ (0, 1). RWR Algorithm can be represented by the following recursive equation:

$$ {p}^{\left(t+1\right)}=\left(1-\theta \right){A}^T{p}^t+\theta {p}^{(0)}, $$

at each step *t*, the RWR algorithm updates the probability *p*^(*t*)^ that the walker is at node *i* at step *t*, until convergence is achieved. Here, *p* represents the probability of each node being a candidate-gene. To initialize the RWR algorithm, we chose 955 genes as seed nodes, which are recognized in the literature as genes differentially expressed between calcified and normal aortic valves [15, 19]. Consequently, the initial probability of being at node *i* was *p*^(0)^ = 1 for 955 seed nodes (prior known genes), while *p*^(0)^ = 1*e* − 05 for the rest of the nodes. The algorithm was repeated until the difference between *p*^(*t* + 1)^ and *p*^(*t*)^ was less than 1*e* − 06. Restart probability equal to *θ* = 0.3 was used, which is the recommended value for PPI networks from the STRING database [12, 20].

### Knowledge-slanted random forest

**Fig. 1:**
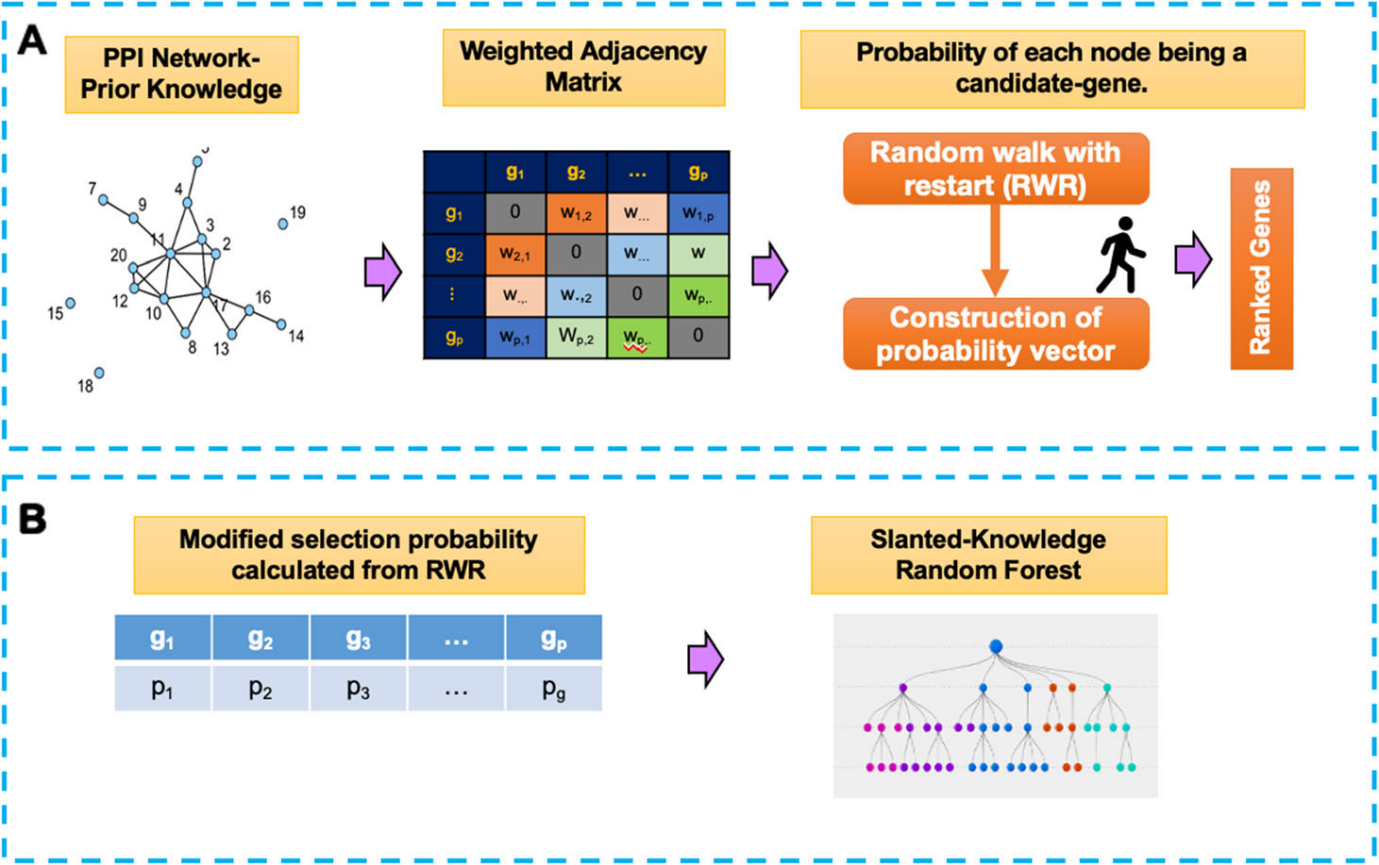
Scheme of the Knowledge-slanted random forest for classification tasks. **A**) The first step is to construct the modified selection probability using the random walk with restart (RWR) over the protein-protein interaction network. **B**) In the second step, a Knowledge-slanted random forest is run using the modified selection probability for each gene

Identification of disease-associated genes was viewed as a classification problem. RF classifier was employed to distinguish gene expression profile of BAV, TAV, and controls from 15,191 genes. RF is a classifier composed of a collection of tree-structure models [21]. The main idea is to sample several data subsets with bootstrap sampling and build a tree in each subset generated. In the conventional RF, for each node within each tree, a subset of features is randomly selected with equal probability and then, the outputs from each model are aggregated by voting from all trees. RF has two parameters: the number of variables available for splitting at each tree node (*mtry*), and the number of trees to grow in each RF (*ntree*). In our knowledge-slanted RF, the selection probability was modified using the probabilities obtained after executing the RWR algorithm with *p*^(*t* + 1)^ that represents the prior knowledge stored in PPI networks. Therefore, the most informative genes can be selected in the first steps of the algorithm. As shown in Fig. 1 this modification allowed the involvement of prior knowledge into RF as an attempt to implement a knowledge-guided supervised learning approach.

We investigated the influence of prior knowledge on the performance of knowledge-slanted RF, randomly selecting 955 genes as seed nodes in RWR to obtain a new probability of selection for each gene. As shown in supplementary Fig. S1, the performance of our approach was similar to that of conventional RF. This confirms that the involvement of prior knowledge including the identification of seed nodes combined with the PPI network from STRING impacts the performance of the RF algorithm.

### Statistical analysis

Continuous variables were summarized with mean ± standard deviation. An RF algorithm using conventional and knowledge-slanted approaches was implemented to distinguish cases of BAV, TAV, and normal valves according to their genetic profile. Initially, the levels of gene expression in cases of BAV, TAV, and normal valves were visualized using T-distributed stochastic neighbor embedding (t-SNE) [22], which is a dimensionality reduction technique that projects the existing relationship in the data from high-dimensional to low-dimensional spaces.

Due to the small sample size (*n* = 27), we used a leave-one-out cross-validation (LOOCV) for tuning parameters in all conventional RF and knowledge-slanted RF. A range of values for *mtry* and *ntree* were swept to evaluate the performance of both methods. The values of *ntree* were ranged from 10 to 1000 trees and *mtry* from 10 to one-third of the number of genes (5064). Comparison between the conventional RF and knowledge-slanted RF was performed with *ntree = 500* and *mtry = 500* when groups were classified into two and three categories. To evaluate the performance several measures were calculated as follows:

- Accuracy = (TP + TN)/ (TP + FN + TN + FP)
- Sensitivity = (TP)/ (TP + FN)
- Specificity = (TN)/(TN + FP)

True positives (TP) are a correct prediction of BAV/TAV cases, true negatives (TN) are a correct prediction of normal valves cases, false negatives (FN) are a false prediction of normal valves among BAV/TAV cases and false positives (FP) are a false prediction of BAV/TAV cases among normal valves cases. All implementations were carried out using the R language and the packages “ranger” [23] and “caret” [24]. Expression levels were compared between groups by a one-way ANOVA test. A *p*-value < 0.05 was considered statistically significant.

## Results

**Fig. 2:**
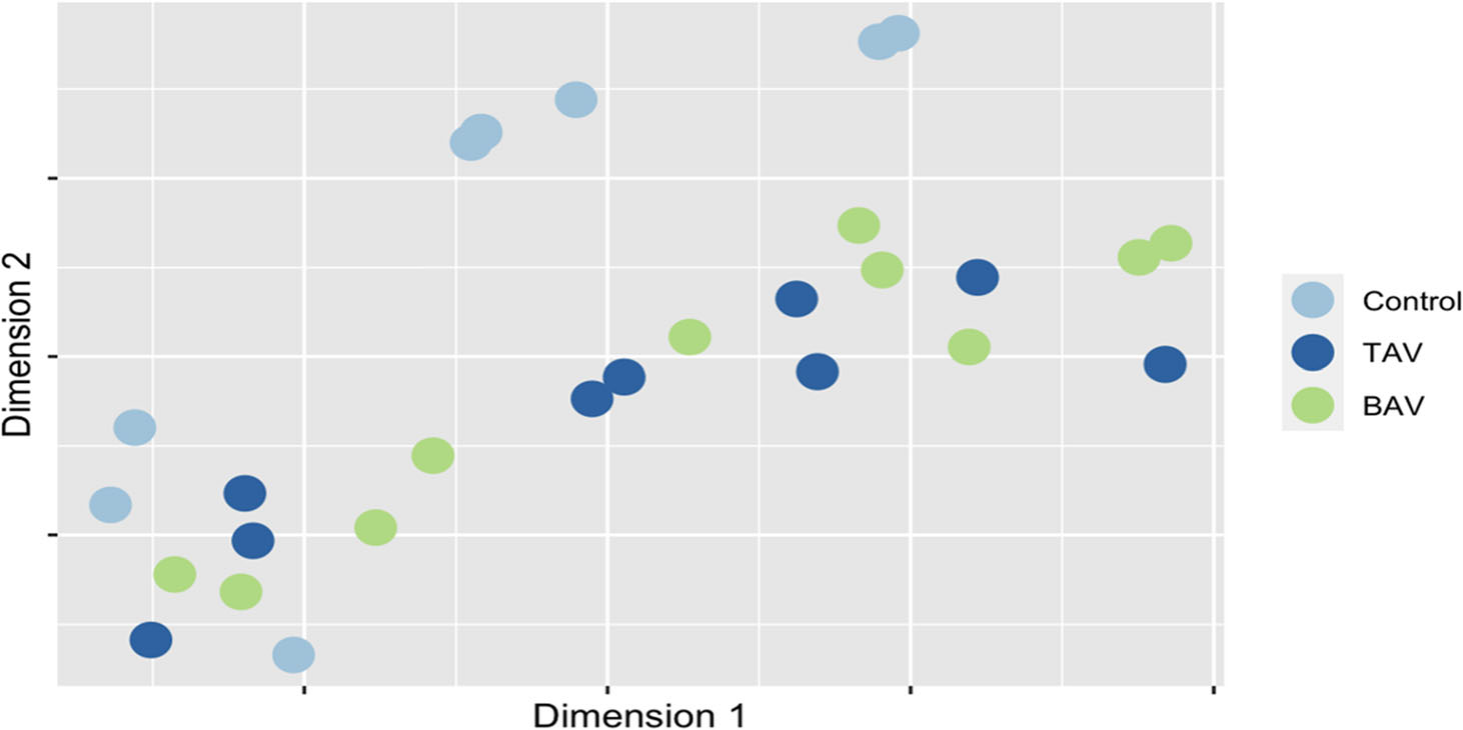
t-SNE visualization for cases with BAV, TAV, and controls

Figure 2 shows the t-SNE plot for the 27 samples classifying the groups into tree CAVS categories (BAV, TAV, and controls). The clustering of sample points evidenced that cases with BAV and TAV share similar level gene expression profiles, with differences compared to the controls.

**Fig. 3:**
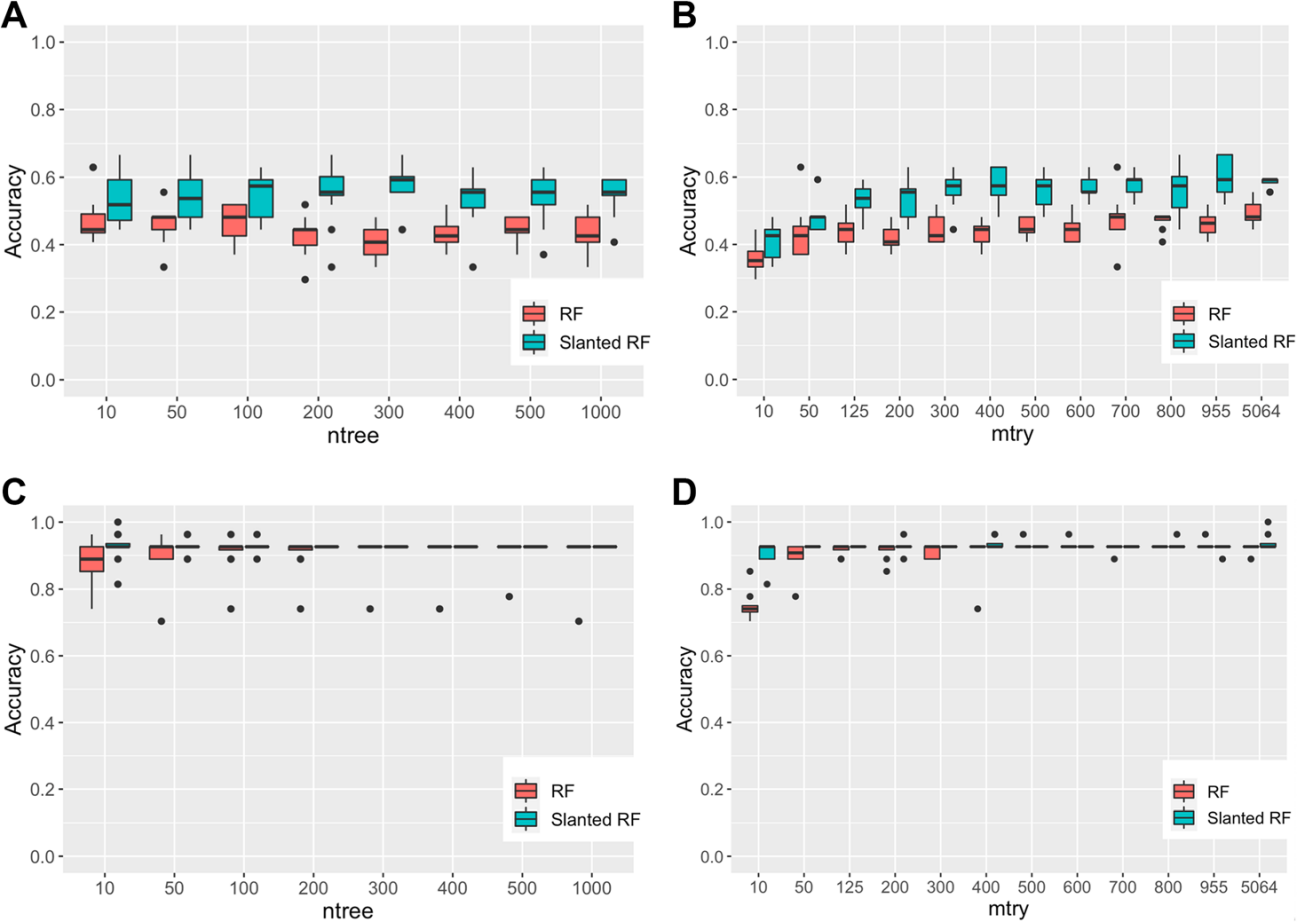
Accuracy performance of knowledge-slanted RF versus conventional RF. **A-B**). Performance for each *ntree* and *mtry* for the identification of genes associated with BAV, TAV, and controls (Three categories). **C-D**). Performance for each *ntree* and *mtry* for the identification of genes associated with BAV or TAV and controls (Two categories)

Knowledge-slanted RF and RF performance measures were compared with and without distinguishing between BAV and TAV patients versus control patients (Fig. 3). The performance of knowledge-slanted RF was better than RF when comparing CAVS patients with BAV or TAV with accuracies of 54.6 ± 6.5 and 43.0% ± 7.1%, respectively. Figure 3A-B evidenced that using the knowledge from a PPI network, *ntree* does not influence the precision of the RF algorithm. While setting the *mtry* parameter with values higher than 400 features (genes), knowledge-slanted RF achieved better performance. When the patients were classified only into two categories (TAV-BAV and controls), knowledge-slanted RF and RF accuracies achieved a mean performance of 92.6% ± 1.8 and 90.3% ± 5.4%, respectively, without differences between both methods. Knowledge-slanted RF even showed better performance when low values of *mtry* = 10 and *ntree* = 10 were used with or without discriminating between BAV and TAV patients (Fig. 3C-D).

After performing LOOCV, the optimal parameters of knowledge-slanted RF and RF were set in *mtry* = 500 and *ntree* = 500. As shown in Table 1, the sensitivity of BAV and TAV cases increased after the inclusion of the information from the PPI network with better overall accuracy and area under the curve. The sensitivity of TAV was lower than that of BAV, conventional RF did not distinguish any TAV cases among all samples and the performance was similar between two-class slanted-RF and RF.

**Table 1:** Performance measures for knowledge-slanted RF and RF algorithms using the optimal model parameters

		Three Categories		Two Categories	
	**CAVS**	**Slanted-RF**	**RF**	**Slanted-RF**	**RF**
Sensitivity	BAV	50.00%	50.00%	100%	100%
	TAV	33.33%	0.00%		
Specificity	BAV	64.70%	47.06%	75.00%	75.00%
	TAV	72.22%	66.67%		
Accuracy	ALL	59.26%	40.74%	92.59%	92.59%
AUC	BAV	0.671	0.589	1.000	1.000
	TAV	0.623	0.451		

CAVS: Calcific aortic valve stenosis; BAV: Bicuspid aortic valve, TAV: Tricuspid aortic valve; RF: Random forest; AUC: Area under the curve

**Fig. 4:**
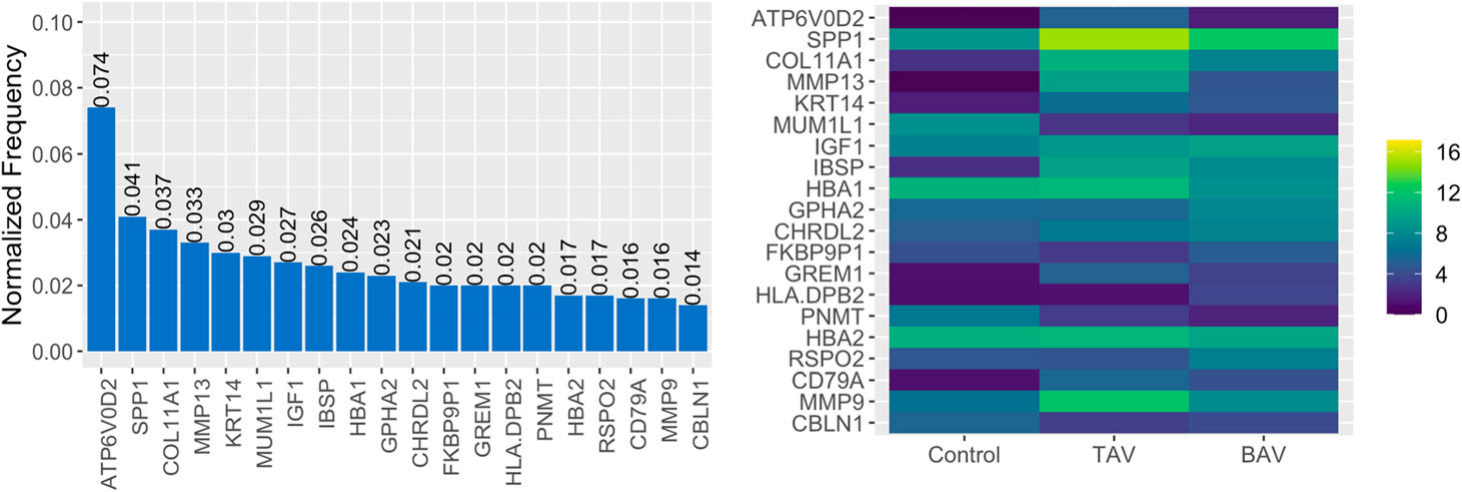
**A**) Frequency of the 20 top genes selected in 500 trees using knowledge-slanted RF. **B**) Normalized Counts comparison (log2) of the 20 top selected genes using knowledge-slanted RF comparing BAV, TAV, and controls patients

To connect the observed performance by knowledge-slanted RF, we calculated the number of times each gene was selected in the 500 trees and compared the normalized counts (log2) in TAV, BAV, and control groups in order to identify the genes associated with CAVS (Fig. 4). Differences in gene expression profiles according to the type of CAVS and patients with normal valves were found (Fig. 4). For example, elevated *ATP6V0D2*, *SPP1, MMP13, KRT14, ISBP, CHRDL2, GREM1,* and *CD79A* were found in CAVS patients compared to controls (*p* < 0.001), while the expression of *MUM1L1*, *PNMT,* and *CBLN1* was lower in the controls. Among 20 of the top genes identified with knowledge-slanted RF, the expression levels of *IGF1* and *RSPO2* were higher in the BAV group than in the TAV group (p < 0.001). We also found that cases with TAV reported higher expression levels of *HLA.DPB2* than the other groups (BAV and controls). The levels of *HBA1*, *HBA2*, *GPHA2,* and *FKBP9P1* were similar between cases with CAVS and normal valves (*p* > 0.05).

## Discussion

In this study, we have introduced the knowledge-slanted RF for classification tasks which integrate the accumulated knowledge in PPI networks into the RF model. Our findings suggest that the knowledge-slanted RF approach reflects prior biological knowledge, leading to improved precision in distinguishing between cases with BAV, TAV, and normal valves. Although cases with BAV and TAV had a similar pattern of gene expression, it is not recommended to combine both groups during any statistical analysis because patients with BAV and TAV have both clinical and imaging differences. For example, Sia et al. showed that patients with TAV compared to BAV have more cardiovascular risk factors, less severe disease, and increased risk of mortality [25].

Interestingly, the main gene used to classify the cases in knowledge-slanted RF was *ATP6V0D2*, which was not reported by Guauque-Olarte et al. [15]. However, Padang et al. [19] had identified that the expression of *ATP6V0D2* was different between cases of BAV with minimal calcification and normal valves, concluding that this gene could be associated with CAVS development in BAV patients. In our samples, no evidence of differences were found in the level expression of *HBA1*, *HBA2*, *GPHA2,* and *FKBP9P1*. However, these genes have been recognized as related genes with CAVS in the literature [19, 26].

In this study, a PPI network was downloaded for the entire gene set (15,191) from STRING and therefore an exhaustive search was carried out requiring a high computational cost due to the large number of genes. The integration of gene interaction data could offer a better prediction performance, specifically when class overlap exists (e.g., TAV vs BAV). In scenarios with easily separable classes (e.g., TAV/BAV vs Control), we believe that the use of prior knowledge would not be useful to achieve better performance because the algorithm can learn directly from the data. For example, in the CAVS dataset among the top-20 most frequent genes, a greater number of shared genes was identified between knowledge-slanted RF and conventional RF when the groups were classified into two categories with 14 shared genes compared to 9 in the multiclass classification (Supplementary Table S1 and S2).

Both knowledge-slanted RF and conventional RF identified associated genes previously recognized in the literature among the top 20 list. However, genes obtained from knowledge-slanted RF ranked better in RWR based on PPI information with a median position of 357.5 compared to 564.5 from conventional RF. This indicates that knowledge-slanted RF could be more easily interpreted by users because the prediction can be attributed mainly to associated genes that could participate in important molecular mechanisms. Additionally, unlike the conventional RF, the knowledge-slanted RF reported three genes (*IGF1, HLA-DPB2, RSPO2*), with a trend towards differential expression levels between BAV and TAV cases (Supplementary Table S3 and S4).

To the best of our knowledge, a couple of approaches that involve prior biological knowledge have been described with respect to the RF algorithm. First, Oskooei et al. [12] considered a Network-based Biased Tree Ensembles (NetBiTE) algorithm for drug sensitivity biomarker identification that involves prior knowledge through a probabilistic bias weight distribution constructed with the information from a biological network using RWR, modifying the probability of selection for each featu1re for splitting a node in RF regression, not for classification tasks. Second, Guan et al. [14] proposed a knowledge-based guided regularized RF (Know-GRRF) that performs a regularized RF using a penalty coefficient for each feature, a score calculated with prior-knowledge obtained from different domains (e.g: published literature) deriving a composite score between 0 and 1 (higher biological relevance). However, the composite score used in the application of Know-GRRF was not computed using the information accumulated in biological networks, which could be considered a limitation.

In contrast to Know-GRRF [14] an advantage of our approach, knowledge-slanted RF, is that it allows the simultaneous analysis of a huge number of features avoiding the implementation of pre-filtering methods for gene prioritization before running knowledge-slanted RF. Similar to Oskooei et al. [12], we show that the RF algorithm when prior knowledge is incorporated to modify the feature selection probability during the construction of tree ensembles outperforms the conventional RF which uses an equal selection probability for each feature.

Among ensemble ML algorithms, RF represents a flexible non-parametric approach with several properties such as invariant to monotonic transformation, robustness to outliers, stability in the presence of correlated variables, or interaction among features [7, 10]. Despite these advantages, in a high-dimensional setting “large P-variables small N-sample size”, RF may provide poor accuracy, especially if complex variable interactions (e.g., gene-gene) exist. Data-driven variable selection methods for classification models based on decision trees have been proposed to minimize the number of input variables (e.g., number of genes) in order to determine the most important predictors and at the same time, achieve more efficient models [27, 28]. However, these approaches do not combine biological prior knowledge with statistical analysis, so the information deposited in biological databases is not used for the prioritization of genes within the models.

Given high-dimensional datasets (*p> > n*) generated in the biological and medical fields, the curse of dimensionality is an inherent problem in analyzing these data, leading to two main effects called data sparsity and distance concentration. Both effects make it more demanding to find similarities and patterns between samples. Although ML techniques have shown better performance in classification tasks on high-dimensional data compared to conventional statistical models [7], ML methods also benefit from feature selection methods to mitigate the curse of dimensionality [29, 30], as we described in this study using a feature ranking strategy.

Despite the encouraging results, this study has some limitations. First, the performance of knowledge-slanted RF could be considered limited. However, the improvement achieved by our approach serves as the basis for complementing the knowledge about CAVS based on valve configuration. Additionally, the number of studies that simultaneously compare cases with BAV and CAV is very small. Second, we do not assess the performance of other ML algorithms. Nevertheless, we evaluated how the use of biological knowledge can improve RF performance in a difficult classification task and a high-dimensional dataset. Third, knowledge-slanted RF was not applied in other datasets and therefore, it cannot be considered as a generalizable approach. However, according to the results found in CAVS dataset, knowledge-slanted RF achieves favorable performance when overlapping classes exist in high dimensional datasets (p*> > n*) and relevant prior biological knowledge about the condition is available.

Future efforts could be focused on evaluating how to involve prior biological knowledge in ML techniques and statistical models and determining whether or not the use of prior knowledge helps to achieve greater model transparency and ease of interpretation using real and simulated datasets.

## Conclusion

In conclusion, the knowledge-slanted RF can outperform RF, especially, when two or more categories share similar characteristics (e.g., gene expression) and discrimination between them could be difficult. In this study, we develop a machine learning guided approach via RF modifying the probability of feature selection according to prior knowledge built with the weights over a protein-protein interaction network. The performance of this proposed approach (knowledge-slanted RF) reported better accuracy in comparison with conventional RF to classify cases with BAV and TAV.

## Supplementary Information

**Additional file 1: Table S1**. Top-20 most frequent genes of Knowledge-slanted RF and conventional RF with three categories. **Table S2**. Top-20 most frequent genes of Knowledge-slanted RF and conventional RF with two categories. **Table S3**. Comparison of the expression levels of the Top-20 most frequent genes identified with knowledge-slanted RF between BAV, TAV and control cases. **Table S4**. Comparison of the expression levels of the Top-20 most frequent genes identified with conventional RF between BAV, TAV and control cases. **Fig. S1**. Accuracy performance of knowledge-slanted RF versus conventional RF when the 955 seed nodes of RWR are selected randomly.

## Data Availability

**CAVS** dataset is available upon request from the corresponding author. Algorithms can be found at https://github.com/ErikaCantor/Knowledge-SlantedRF.

## Abbreviations

CAVS: Calcific aortic valve stenosis
BAV: Bicuspid aortic valve
TAV: Tricuspid aortic valve
RF: Random forest
ML: Machine learning
RWR: Random walk with restart
PPI: protein-protein interactions
