# Supplementary material for: Biological knowledge-slanted random forest approach for the classification of calcified aortic valve stenosis
Source: BioData Min. 2021 Jul 23;14:35. doi: 10.1186/s13040-021-00269-4 (PMC8305490; doi:10.1186/s13040-021-00269-4)

Supplementary Material - Biodata Mining

Biological knowledge-slanted random forest approach for the classification of calcified aortic valve stenosis

Erika Cantor[1], Rodrigo Salas[2], Harvey Rosas[3], Sandra Guauque-Olarte[4]

[1]Institute of Statistics, Universidad de Valparaiso, Valparaiso, Chile. E-mail: erika.cantor@postgrado.uv.cl. [2]School of Biomedical Engineering, Universidad de Valparaiso, Valparaiso, Chile. E-mail: Rodrigo.salas@uv.cl. [3]Institute of Statistics, Universidad de Valparaiso, Valparaiso, Chile. E-mail: harvey.rosas@uv.cl. [4]Faculty of Dentistry, Universidad Cooperativa de Colombia, Medellín, Colombia. E-mail: sandra.guauque@campusucc.edu.co

Table S1. Top-20 most frequent genes of Knowledge-slanted RF and conventional RF with three categories

Top	Knowledge-slanted RF			Conventional RF		
	**Rank RWR**	**Gene**	**Gene from Literature**	**Rank RWR**	**Gene**	**Gene from Literature**
1	643	ATP6V0D2	Yes	148	KRT14	Yes
2	47	SSP1	Yes	49	COL11A1	Yes
3	49	COL11A1	Yes	643	ATP6V0D2	Yes
4	119	MMP13	Yes	199	IBSP	Yes
5	148	KRT14	Yes	4652	CLEC4G	No
6	790	MUM1L1	Yes	119	MMP13	Yes
7	20	IGF1	Yes	47	SPP1	Yes
8	199	IBSP	Yes	707	IL21R	Yes
9	441	HBA1	Yes	357	PNMT	Yes
10	358	GPHA2	Yes	687	RPRML	Yes
11	675	CHRDL2	Yes	4405	HS6ST2	No
12	1068	FKBP9P1	Yes	486	GREM1	Yes
13	486	GREM1	Yes	910	CBLN4	Yes
14	113	HLA.DPB2	Yes	665	TDO2	Yes
15	357	PNMT	Yes	4326	GRID2	No
16	490	HBA2	Yes	409	SP7	Yes
17	718	RSPO2	Yes	165	COL10A1	Yes
18	70	CD79A	Yes	88	FCGR3A	Yes
19	27	MMP9	Yes	790	MUM1L1	Yes
20	536	CBLN1	Yes	14596	SIGLEC16	No

RF: Random Forest, RWR: random walk with restart

Table S2. Top-20 most frequent genes of Knowledge-slanted RF and conventional RF with two categories

Top	Knowledge-slanted RF			Conventional RF		
	**Rank RWR**	**Gene**	**Gene from Literature**	**Rank RWR**	**Gene**	**Gene from Literature**
1	199	ISBP	Yes	49	COL11A1	Yes
2	49	COL11A1	Yes	148	KRT14	Yes
3	148	KRT14	Yes	199	IBSP	Yes
4	47	SPP1	Yes	707	IL21R	Yes
5	790	MUM1L1	Yes	119	MMP13	Yes
6	119	MMP13	Yes	47	SPP1	Yes
7	486	GREM1	Yes	88	FCGR3A	Yes
8	357	PNMT	Yes	357	PNMT	Yes
9	165	COL10A1	Yes	687	RPRML	Yes
10	910	FCGR3A	Yes	4405	HS6ST2	No
11	665	TDO2	Yes	409	SP7	Yes
12	910	CBLN4	Yes	486	GREM1	Yes
13	70	CD79A	Yes	165	COL10A1	Yes
14	707	IL21R	Yes	910	CBLN4	Yes
15	7	LCK	Yes	790	MUM1L1	Yes
16	146	TNFSF11	Yes	592	TMEM200A	Yes
17	67	NPY	Yes	67	NPY	Yes
18	510	SLAMF7	Yes	286	CXCL5	Yes
19	409	SP7	Yes	600	DIRAS1	Yes
20	749	GPR68	Yes	915	LRRC15	Yes

RF: Random Forest, RWR: random walk with restart

Table S3. Comparison of the expression levels of the Top-20 most frequent genes identified with knowledge-slanted RF between BAV, TAV and control cases.

Gene	Control (n=8)	TAV (n=9)	BAV (n=10)	All	Control vs TAV	Control vs BAV	TAV vs BAV
ATP6V0D2	0.79 ± 0.80	4.03 ± 0.98	3.13 ± 1.35	0.000	0.000	0.000	0.252
SPP1	9.37 ± 1.60	14.14 ± 1.36	13.76 ± 1.04	0.000	0.000	0.000	1.000
COL11A1	3.65 ± 1.17	8.36 ± 1.29	8.11 ± 1.20	0.000	0.000	0.000	1.000
MMP13	0.21 ± 0.58	5.44 ± 2.27	4.55 ± 1.96	0.000	0.000	0.000	0.889
KRT14	1.98 ± 1.07	6.80 ± 1.24	7.64 ± 1.72	0.000	0.000	0.000	0.601
MUM1L1	6.82 ± 2.17	2.86 ± 1.26	3.17 ± 0.82	0.000	0.000	0.000	1.000
IGF1	8.61 ± 0.96	8.98 ± 0.82	9.87 ± 0.52	0.006	0.983	0.006	0.058
IBSP	1.93 ± 1.53	8.99 ± 1.05	8.51 ± 1.30	0.000	0.000	0.000	1.000
HBA1	10.42 ± 1.37	10.26 ± 1.06	9.38 ± 1.00	0.128			
GPHA2	7.02 ± 1.81	6.74 ± 1.58	8.32 ± 1.13	0.070			
CHRDL2	4.92 ± 1.76	7.27 ± 1.34	8.39 ± 0.95	0.000	0.005	0.000	0.254
FKBP9P1	5.18 ± 1.58	4.00 ± 0.68	5.07 ± 1.18	0.089			
GREM1	2.16 ± 1.00	4.73 ± 1.25	4.57 ± 0.98	0.000	0.000	0.000	1.000
HLA.DPB2	2.15 ± 1.34	4.55 ± 1.58	2.96 ± 1.22	0.752	0.005	0.683	0.060
PNMT	6.35 ± 1.42	2.92 ± 0.90	2.80 ± 0.97	0.000	0.000	0.000	1.000
HBA2	11.02 ± 1.21	10.95 ± 0.91	10.21 ± 0.95	0.181			
RSPO2	4.98 ± 0.99	5.87 ± 0.70	6.94 ± 0.82	0.000	0.114	0.000	0.033
CD79A	2.54 ± 0.76	6.24 ± 2.32	5.83 ± 1.18	0.001	0.000	0.001	1.000
MMP9	5.80 ± 2.48	10.10 ± 1.60	9.08 ± 1.57	0.000	0.000	0.004	0.752
CBLN1	5.64 ± 1.04	3.33 ± 0.59	3.88 ± 0.99	0.000	0.000	0.001	0.560

BAV: Bicuspid aortic valve; TAV: Tricuspid aortic valve

Table S4. Comparison of the expression levels of the Top-20 most frequent genes identified with conventional RF between BAV, TAV and control cases.

**Gene**	**Control (n=8)**	**TAV (n=9)**	**BAV (n=10)**	**All**	**Controls vs TAV**	**Control vs BAV**	**TAV vs BAV**
KRT14	1.98 ± 1.07	6.80 ± 1.24	7.64 ± 1.72	0.000	0.000	0.000	0.601
COL11A1	3.65 ± 1.17	8.36 ± 1.29	8.11 ± 1.20	0.000	0.000	0.000	1.000
ATP6V0D2	0.79 ± 0.80	4.03 ± 0.98	3.13 ± 1.35	0.000	0.000	0.000	0.252
IBSP	1.93 ± 1.53	8.99 ± 1.05	8.51 ± 1.30	0.000	0.000	0.000	1.000
CLEC4G	6.38 ± 1.20	7.65 ± 0.45	8.30 ± 1.46	0.006	0.091	0.005	0.674
MMP13	0.21 ± 0.58	5.44 ± 2.27	4.55 ± 1.96	0.000	0.000	0.000	0.889
SPP1	9.37 ± 1.60	14.14 ± 1.36	13.76 ± 1.04	0.000	0.000	0.000	1.000
IL21R	2.68 ± 0.99	5.82 ± 0.90	6.02 ± 1.11	0.000	0.000	0.000	1.000
PNMT	6.35 ± 1.42	2.92 ± 0.90	2.80 ± 0.97	0.000	0.000	0.000	1.000
RPRML	3.61 ± 1.44	5.34 ± 1.08	5.48 ± 0.78	0.003	0.011	0.005	1.000
HS6ST2	3.92 ± 0.84	1.29 ± 0.98	1.21 ± 0.90	0.000	0.000	0.000	1.000
GREM1	2.16 ± 1.00	4.73 ± 1.25	4.57 ± 0.98	0.000	0.000	0.000	1.000
CBLN4	0.24 ± 0.44	4.43 ± 1.37	4.74 ± 1.02	0.000	0.000	0.000	1.000
TDO2	0.32 ± 0.60	3.89 ± 1.04	3.84 ± 1.15	0.000	0.000	0.000	1.000
GRID2	3.18 ± 1.54	2.69 ± 1.05	1.68 ± 2.74	0.030	1.000	0.032	0.205
SP7	1.17 ± 0.72	4.26 ± 1.53	4.36 ± 1.07	0.000	0.000	0.000	1.000
COL10A1	2.12 ± 1.38	7.04 ± 1.43	7.06 ± 1.05	0.000	0.000	0.000	1.000
FCGR3A	9.04 ± 1.09	11.19 ± 1.14	11.41 ± 1.15	0.000	0.002	0.001	1.000
MUM1L1	6.82 ± 2.17	2.86 ± 1.26	3.17 ± 0.82	0.000	0.000	0.000	1.000
SIGLEC16	2.53 ± 0.92	3.29 ± 0.97	2.65 ± 0.66	0.148			

BAV: Bicuspid aortic valve; TAV: Tricuspid aortic valve

Figure S1. Accuracy performance of knowledge-slanted RF versus conventional RF when the 955 seed nodes of RWR are selected randomly.

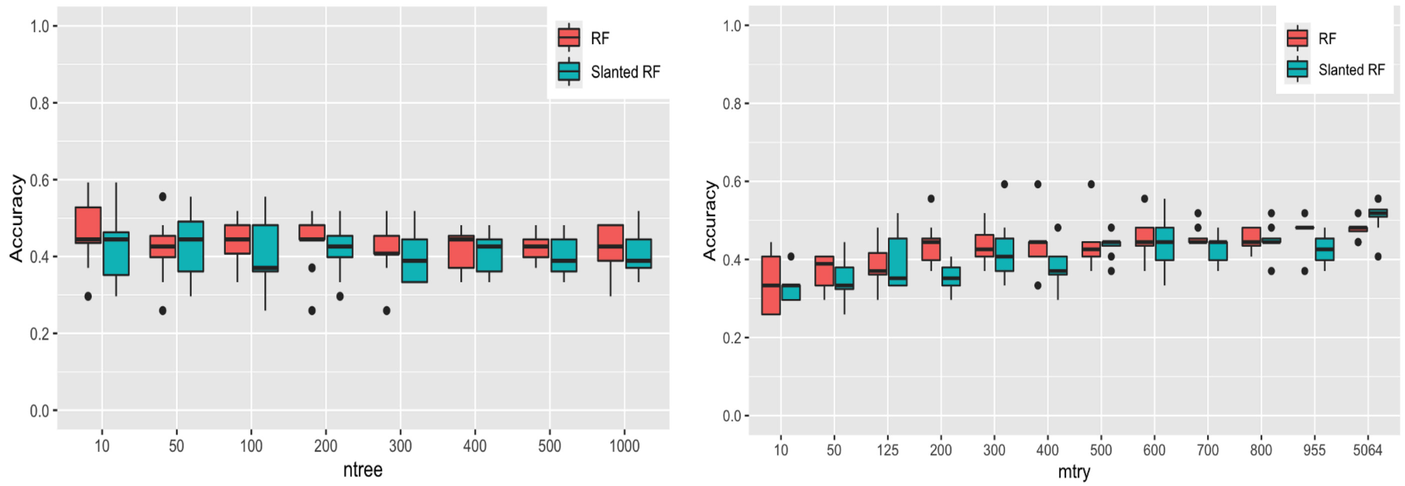

Supplement: Supplementary file 1 — Additional file 1: Table S1. Top-20 most frequent genes of Knowledge-slanted RF and conventional RF with three categories. Table S2. Top-20 most frequent genes of Knowledge-slanted RF and conventional RF with two categories. Table S3. Comparison of the expression levels of the Top-20 most frequent genes identified with knowledge-slanted RF between BAV, TAV and control cases. Table S4. Comparison of the expression levels of the Top-20 most frequent genes identified with conventional RF between BAV, TAV and control cases. Fig. S1. Accuracy performance of knowledge-slanted RF versus conventional RF when the 955 seed nodes of RWR are selected randomly. [file 13040_2021_269_MOESM1_ESM.pdf]
